# Role of T cells in severe COVID-19 disease, protection, and long term immunity

**DOI:** 10.1007/s00251-023-01294-9

**Published:** 2023-02-08

**Authors:** Julia Maret Hermens, Can Kesmir

**Affiliations:** Theoretical Biology and Bioinformatics, Biology Department, Science Faculty, Utrecht University, Utrecht, Netherlands

**Keywords:** T cell immunity, SARS-CoV-2, Vaccination, Protection against severe COVID-19

## Abstract

Infection with SARS-CoV-2 causes wide range of disease severities from asymptomatic to life-threatening disease. Understanding the contribution of immunological traits in immunity against SARS-CoV-2 and in protection against severe COVID-19 could result in effective measures to prevent development of severe disease. While the role of cytokines and antibodies has been thoroughly studied, this is not the case for T cells. In this review, the association between T cells and COVID-19 disease severity and protection upon reexposure is discussed. While infiltration of overactivated cytotoxic T cells might be harmful in the infected tissue, fast responding T cells are important in the protection against severe COVID-19. This protection could even be viable in the long term as long-living memory T cells seem to be stabilized and mutations do not appear to have a large impact on T cell responses. Thus, after vaccination and infections, memory T cells should be able to help prevent onset of severe disease for most cases. Considering this, it would be useful to add N or M proteins in vaccinations, alongside the S protein which is currently used, as this results in a broader T cell response.

## Introduction

Since the emergence of severe acute respiratory syndrome coronavirus 2 (SARS-CoV-2), over 6 million deaths and almost 500 million infections, as a result of this novel virus, have been reported (World Health Organization 2022). Worldwide research was promptly undertaken to discover more about SARS-CoV-2 and the corresponding disease COVID-19. In little over 2 years since the emergence of the virus, a wide array of knowledge has been gathered. Individuals suffering from the corresponding disease (COVID-19) can be divided into five groups, based on disease severity: asymptomatic, mild, moderate, severe, and critical (Gao et al. 2021). Severe disease complications observed in a subset of COVID-19 patients include pneumonia, acute respiratory distress syndrome (ARDS) and cardiac injury (Huang et al. 2020).

Large collections of data from patients with mild and severe disease allowed identifying several risk factors for severe COVID-19. These factors include age, sex, and comorbidities such as cardiovascular disease, diabetes, and obesity (Huang et al. 2020). The magnitude of initial innate immune responses also affects the disease outcome: high levels of cytokines (cytokine storm) are observed in individuals with severe disease (Jacob 2020; Wang et al. 2020a). The potential impact of the adaptive immune system on pathology or protection is often deemed trivial in comparison (Jacob 2020). However, this is not necessarily true. Even though GWAS studies have not located any SNP associated with disease severity on the HLA loci (Pairo-Castineira et al. 2021; Shelton et al. 2021), this does not exclude that the adaptive immune responses might play an important role. The adaptive immune response is already active prior to hospitalization, around 7 days post-symptom onset (Huang et al. 2020), which suggests that the adaptive immune system could contribute to disease severity. Furthermore, the abovementioned risk factors have direct effects on both innate and adaptive immune responses. In males, poor CD8 T cell activation was correlated with worse disease outcome, while worse disease outcome in females was associated with higher levels of innate immune cytokines (Brodin 2021; Takahashi et al. 2020). Thus, the adaptive immune responses, or at least T cells, should be taken into account when studying COVID-19 severity.

Clearly, the development of long-term immunity, and thus the ability of the adaptive immune system to develop and retain memory responses, is crucial to minimize health problems caused by SARS-CoV-2 infection. The longevity of functional immunity depends partially on the mutability of the virus. Despite the presence of an essential protein associated with RNA proofreading (Denison et al. 2011; Ogando et al. 2020; Smith et al. 2014), the mutation rate of SARS-CoV-2 is still relatively high (Safari and Elahi 2022) and multiple new variants of SARS-CoV-2, such as Alpha, Beta, Gamma, Delta, and Omicron, have been observed so far. The variants with a strong potential to increase their transmissibility, pathogenicity, severity of clinical symptoms, escape from treatment, and evasion from the immune system have been denoted as variants of concern (VOCs) (Mistry et al. 2022; Safari and Elahi 2022). The new variants mainly, but not solely, contain mutations in the Spike (S) protein. Most of the mutations that increase viral fitness occur within the receptor binding domain (RBD) of this protein, which binds the ACE2 protein on host cells to mediate cell entry (Mistry et al. 2022). Thus far, B cells and antibodies have often been the focus of studies on immunity. The current vaccines are specifically developed to produce an antibody response against the S protein of SARS-CoV-2, due to its importance in cell entry. Unfortunately, many antibody responses seem to be short lasting, as was the case for SARS-CoV-1 in which the majority of studied subjects did not have detectable levels of SARS-CoV-1-specific IgG antibodies or memory B cells 6 years after infection, while memory T cells were observed in most of the recovered patients (Tang et al. 2011). Furthermore, the ability of antibodies to respond to the VOCs also significantly deteriorates (Geers et al. 2021; Zhan et al. 2021) which shows, again, that antibodies will most likely not be protective over a long period of time, even when they are still present.

More and more evidence surfaces on the crucial role of T cells (Hossain et al. 2021; Mistry et al. 2022). One of such studies focussed on two hospitalized COVID-19 patients who recovered despite X-linked agammaglobulinemia, a condition where the patients do not have any B cells in their peripheral blood. This result suggests the humoral response was not necessary to survive the infection, even though the absence of this B cell response might have contributed to more severe symptoms during COVID-19. Another immune mechanism such as the T cell response, as the authors’ state, might have helped these patients overcome the disease (Soresina et al. 2020). The hypothesized role of T cells is reinforced by the observation that some individuals who remain seronegative after recovery have SARS-CoV-2-specific T cell responses (Sekine et al. 2020).

Given these observations, it is necessary to specifically look at the (long term) role of T cells in disease severity. This could give more insight in recovery and pathology of individuals infected with SARS-CoV-2 and therefore drive prospective modes of treatment and vaccination strategies against disease caused by both SARS-CoV-2 and other novel pathogens. This review, therefore, will discuss the current knowledge on the contribution of T cells in the protection against disease progression, and in immunity for SARS-CoV-2. We will focus on three questions: Do T cells contribute or counteract disease progression after infection with SARS-CoV-2? Does developed T cell immunity protect against severe disease upon reinfection? Is T cell immunity still effective in the long term?

## Role of T cells in disease severity

One of the best known recorded symptoms observed in hospitalized COVID-19 patients is lymphopenia in the blood (Huang et al. 2020). The absolute number of CD4 and CD8 T cells was decreased within all of the studied COVID-19 patients compared to healthy individuals. The decline in T lymphocytes was especially strong within the more severe cases (Chen et al. 2020; Liu et al. 2020; Sekine et al. 2020; Wang et al. 2020b; Xu et al. 2020). In hospitalized COVID-19 patients, an increase in the CD8 T cells, and in some studies also in the CD4 T cells, was strongly associated with a successful treatment. No significant increase in T cell numbers was observed in failed treatments (Rezaei et al. 2021; Wang et al. 2020b). These observations show that the amount of T cells in the blood is associated with disease outcome.

One of the explanations for lymphopenia in the blood is relocation of cells to inflamed lung tissue (Jafarzadeh et al. 2021; Wang et al. 2020b). Infiltration of T cells within inflamed tissue and markers for migration has indeed been observed in subjects who died due to severe COVID-19 (Adamo et al. 2021; Gauchotte et al. 2021; Wichmann et al. 2020; Xu et al. 2020). However, within bronchoalveolar lavage fluid (BALF), the percentage of CD8 T cells in severe patients was decreased compared to those with moderate disease (Liao et al. 2020). These results suggest that there might be multiple other mechanisms that could account for lymphopenia, such as cell death of the lymphocytes (Jafarzadeh et al. 2021), which is corroborated by the increase of apoptosis markers found in COVID-19 patients (André et al. 2022). The amount of observed T cells with apoptotic marks was even associated with disease severity (Adamo et al. 2021).

Several studies have noted enrichment of activated T cells (CD38 + HLA-DR +) in severe COVID-19 patients (Du et al. 2021; Georg et al. 2022; Mathew et al. 2020). This subset might represent three groups of T cells: overactivated, exhausted, or apoptosis-sensitive cells (Du et al. 2021). In line with this, other markers expressed by these cells (e.g., PD-1, Tim-3, and CD94/NKG2A) have also been associated with disease progression (Diao et al. 2020; Zheng et al. 2020). In influenza patients, the majority of CD38 + HLA-DR + CD8 cells expressed PD-1 (Wang et al. 2018): after recovery, all activated CD8 cells had intermediate levels of PD-1 expression, while cells with both intermediate and high expression of PD-1 were present within fatally ill patients. As influenza in many ways causes a similar infection in humans as SAR-CoV-2, the PD-1 marker observed in COVID-19 patients are most likely accumulated during T cell (over)activation, probably as a result of high viral loads. These PD-1-expressing CD8 T cells are shown to be still functional within COVID-19 patients (Rha et al. 2021).

Bergamaschi et al. (2021) studied the activated CD8 T cells (CD8 + HLA-DR + CD38 + T cells) over time in subjects differing in disease severity. Several interesting findings came out of this study. First of all, the increase in activated CD8 T cells occurred later in patients with severe disease compared to patients with mild disease. Second, in both the asymptomatic and mild disease groups, the number of activated CD8 T cells contracted after the first 2 weeks post-disease onset, while these cells kept on increasing to much higher levels within the severely ill patient group (Bergamaschi et al. 2021). In a different cohort, Xu et al. (2021) looked at different CD8 T cell subsets and showed that a delayed increase in CD8 effector memory was associated with severe disease as well. The association of severe disease outcome with high numbers of overactivated CD8 T cells from week two onwards has been observed before (Zeng et al. 2020), but the early protective increase of activated CD8 T cells has not been observed in all studies (Georg et al. 2022). The results of Bergamaschi et al. (2021) are in agreement with influenza cases: in influenza, an early rise in activated CD8 T cells (CD38 + HLA-DR + PD-1 +) was also associated with survival while these cells persisted in fatally ill patients (Wang et al. 2018). The importance of a fast response also indicated the essential role of memory T cells in influenza (Wang et al. 2015). All in all, current studies suggest that early CD8 T cell responses are protective. Whether the increase in these cells contribute to disease severity or are simply a side effect of persistent disease should be studied further.

Next question is whether or not the early activated T cells in the first weeks post-symptom onset are SARS-CoV-2 specific? Bergamachi et al. (2021) found no difference in the number of SARS-CoV-2-specific T cells between mild and severe cases during the first and or second week. This suggests that the early increase in CD8 T cells in individuals with mild disease is caused by non-specific, bystander, activated T cells (Bergamaschi et al. 2021). Bystander activation of CD8 T cells occurs in the presence of pro-inflammatory cytokines, before antigen-specific CD8 T cells are activated. Therefore, the bystander-activated cells can respond faster than the specific adaptive immune response (Maurice et al. 2021). The presence of the bystander T cells was further supported by the elevated levels of bystander activation markers, NKG2D and IL-7R, within subjects with mild disease. Due to activation of bystander CD8 T cells, individuals with asymptomatic and mild disease have an early adaptive immune response before antigen-specific responses are present. These results suggest that bystander-activated CD8 T cells could prevent disease progression via (non-specific) viral clearance before the start of symptoms (Bergamaschi et al. 2021).

### Pathology due to overactivated CD8 T cells

The continuous rise of overactivated CD8 T cells in severely ill patients suggests a potential contribution of these cells in disease progression. Indeed, the overactivated CD38hi CD8 T cells have been correlated with cytokine storm and myocardial, renal, and liver injury in severe COVID-19 patients (Du et al. 2021). Moreover, highly cytotoxic T cells were present within damaged organs of deceased patients (Gauchotte et al. 2021), suggesting, again, a potential association between increased cytotoxicity and tissue damage and lethal disease outcome (Szabo et al. 2021; Xu et al. 2020). However, this does not yet prove that the T cells are directly involved in the tissue damage.

**Fig. 1:**
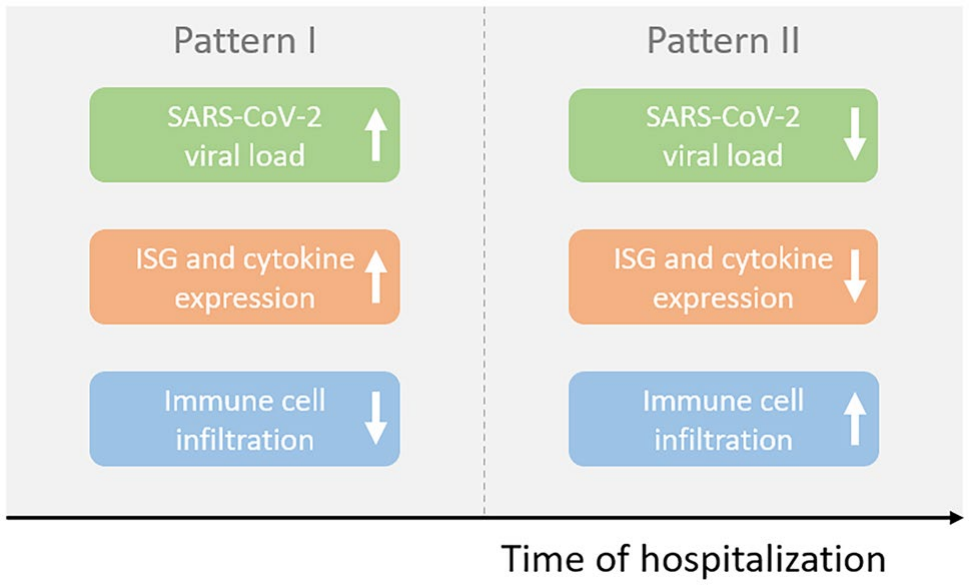
Two distinct patterns, observed by Nienhold et al. (2020), in deceased COVID-19 patients. Early on (pattern I) high ISG expression, high viral load, and little infiltration of lymphocytes are observed. Later on in the disease (pattern II) ISG expression and viral load are lower, there is a high infiltration of immune cells such as lymphocytes. Deceased patients fall in one of these two patient groups

Nienhold et al. (2020) showed that there are two different patterns that can be distinguished within lethal COVID-19 patients (Fig. 1). Patients who died relatively soon after hospitalization displayed a pattern of high viral load, little tissue damage within lungs and high local expression of interferon stimulated genes (ISGs) and cytokines (pattern I). Patients who died after a longer stay in the hospital had lower viral load, but lung injury, low ISG expression, and a lot of infiltration of activated CD8 T cells (pattern II). The number of CD8 T cells within lung tissue increases over time, where they can contribute to alveolar tissue damage and pneumonia symptoms (Nienhold et al. 2020). However, a number of studies demonstrate that innate immune cell infiltration is the main cause of tissue damage in severe SARS-CoV-2. In one of these studies, CD8 T cells were increased in patients with ARDS, not due to SARS-CoV-2 infection, but as a result of bacterial pneumonia (Rendeiro et al. 2021).

Bystander-activated CD8 T cells can, as well as specific cells, potentially contribute to the tissue damage (Bergamaschi et al. 2021). Previously, the role of TCR-independent T cells in tissue damage has been suggested in celiac disease and HAV infection (Lee et al. 2022). In line with this, bystander-activated CD8 T cells have also been observed within the blood of severely ill SARS-CoV-2 patients in the ICU (Gregorova et al. 2020). Adamo et al. (2022) show that there were more non-specific CD8 T cells (Cov-2Dex-) in severe patients than in mild disease patients. This, again, suggested that higher bystander activation was present in patients with severe disease. It is possible that a substantial amount of the bystander cells become activated after the onset of the cytokine storm. Indeed, some of the cytokines increased in ICU patients (Huang et al. 2020) have the capability to activate both CD4 and CD8 T cells independent of the presence of antigens (Unutmaz et al. 1994; Wong and Pamer 2001). This corresponds to the second pattern described by Nienhold et al. (2020) where increase in immune infiltration, and therefore activated T cells, is delayed compared to the increase of cytokines. Furthermore, some of the cytokines present in COVID-19 patients might increase the expression of PD-1 on bystander-activated cells (Kinter et al. 2008). This could partially account for the increase in potentially overactivated T cells in severely ill patients. It should however be noted that further understanding of bystander activation is needed before definite conclusions about the detrimental effect of this mechanism can be made (Lee et al. 2022).

In addition to activated bystander and specific CD38 high CD8 T cells, other cytotoxic T cells can be activated simply because of interactions of these cells with other immune components. For example, an increased complement deposition of especially C3a, as found in the lungs of deceased patients, could further increase lung damage by activating CD16 + cytotoxic T cells (Nienhold et al. 2020). These cells indeed further contribute to endothelial injury and fatal disease outcome (Georg et al. 2022). All in all, multiple different mechanisms activate cytotoxic T cells during COVID-19 and might partially account for the observed tissue damage in some patients. More in-depth studies should focus on the presence of a causal relationship.

Besides causing direct tissue damage, the T cells can also contribute to disease severity indirectly by producing cytokines. Some patients with COVID-19, including children with multisystem inflammatory syndrome (MIS-C), show a similar disease pattern (e.g., persistent fever and hyperinflammation in multiorgan systems, etc.) as seen by patients with toxic shock syndrome (TSS) (Cheng et al. 2020; Porritt et al. 2021), which is caused by direct binding of superantigens to TCRs or MHC class II molecules, which activates T cells (Krakauer 2019). The activation of T cells via superantigens could theoretically cause a cytokine storm. In SARS-CoV-2, a spike motif closely resembles a bacterial superantigen (Cheng et al. 2020; Porritt et al. 2021). Patients with hyperinflammatory COVID-19 show expansion of TCRs that corresponds with the superantigen selection process one would expect to see in TSS. Moreover, the TCRs enriched in severe COVID-19 patients were able to bind the superantigen-like motif of the SARS-CoV-2 spike protein (Cheng et al. 2020). Taken together, this data suggests that continuous activation of T cells in SARS-CoV-2 infection could contribute to lung damage either by direct cytotoxic action and/or by increasing local cytokine concentrations.

## Role of T cells in protection

The presence of SARS-CoV-2-specific memory T cells after natural infections has been studied by stimulating T cells from blood samples of recovered individuals with SARS-CoV-2 antigens. Several studies found that most patients had (~ 40–86 days post-disease onset) a T memory response (CD4 and CD8) against at least one of the peptides from the SARS-CoV-2 proteins S, M, N, E, and ORF1ab (Peng et al. 2020; Tavukcuoglu et al. 2021; Wang et al. 2021). Sekine et al. (2020) and Wang et al. (2021) show that SARS-CoV-2-specific T cell responses were not only present in symptomatic patients. Asymptomatic patients and seronegative exposed individuals also had a significantly higher T cell response compared to healthy controls (Wang et al. 2021). Tavukcuoglu et al. (2021) studied the T memory response in symptomatic patients further. They showed that both central memory ($T_{CM}$) and effector memory (${T}_{EM}$) cells had increased proliferation capacity and had a functional response in the majority of the symptomatic COVID-19 patients. The highest proliferation capacity was observed for CD4 ${T}_{CM}$ cells although the results were very variable between patients (Tavukcuoglu et al. 2021). Peng et al. (2020) also found that the highest percentage of CD8 T cell response was CD8 ${T}_{EM}$ cells when peptides of all SARS-CoV-2 proteins except for ORF1 were taken into account. Rodda et al. (2021) performed a similar study in which CD4 ${T}_{CM}$, and CD4 ${T}_{EM}$ to a lesser extent, showed substantial proliferation upon stimulation with peptides from S protein. These results thus show that circulating ${T}_{CM}$ cells and ${T}_{EM}$ cells are developed in naturally infected individuals and keep their ability to proliferate upon stimulation. Therefore, a memory response could activate upon reinfections to ensure rapid clearance and better disease course.

Besides circulating memory T cells, the tissue resident memory (${T}_{RM}$) cells are also important factors in generating protective immune responses. These cells are able to respond relatively fast upon infection, because they are already present at the location of viral entry. However, ${T}_{RM}$ cells will most likely not be detected in studies that focus on the peripheral compartment as they do not circulate, but are permanent residents of tissue (Martin and Badovinac 2018). Therefore, it is also important to infer the presence of SARS-CoV-2-specific cells from studies that specifically looked at the compartments of the respiratory system. Roukens et al. (2022) studied samples from hospitalized patients that corresponded to 11–82 days post-symptom onset, while Grau-Expósito et al. (2021) looked at asymptomatic, mild symptomatic, and severe symptomatic patients over a longer period of time (up to 10 months for a subset of samples). Both studies show that ${T}_{RM}$ cells develop in the respiratory compartment, while these cells could generally not be identified in the peripheral compartment (Grau-Expósito et al. 2021). Approximately 60% of the total nasal CD8 T cells were of ${T}_{RM}$ phenotype (Roukens et al. 2022), and for the SARS-CoV-2 specific CD4 and CD8 T cells, there was also a trend that ${T}_{RM}$ cells were dominant (Grau-Expósito et al. 2021). Furthermore, the percentage of CD8 cells was higher in both convalescent and healthy subjects compared to CD4 cells.

Above results clearly demonstrate that ${T}_{RM}$ cells in the respiratory compartment, a good indication of long lasting immunity, develop after SARS-CoV-2 infection. Is the development of memory T cells, and therefore immunity, affected by disease course? Peng et al. (2020) studied memory T cell responses against SARS-CoV-2 antigens, in recovered mild and severe COVID-19 patients, ~ 40 days post-symptom onset. Even though lymphopenia is associated with more severe disease, the overall T cell response, especially against S, M, ORF3, and ORF8 proteins, was broader and higher for severe patients (Peng et al. 2020). The observed higher T cell response could simply be due to a longer and more elaborate local immune response in severely ill patients. Sekine et al. (2020) compared a wider range of disease severity (patients with mild or severe disease, as well as exposed family members). The height of response, against peptides of the S, M, and N proteins, was again positively correlated with disease severity (Sekine et al. 2020). When the T cell subsets were considered separately, a more nuanced result was obtained. The CD8 T cell response against S, M, and N was higher in mild patients compared to severe patients (Peng et al. 2020) and severe patients had a higher total frequency of CD4 ${T}_{EM}$ and lower frequency of CD4 ${T}_{CM}$ cells compared to patients with milder disease course (Gong et al. 2020). All in all, these results suggest that memory T cells are generated over a wide scale of disease severity and that their amounts depend on the disease severity.

Due to the possible adverse outcome of infection with SARS-CoV-2 (Huang et al. 2020), the use of vaccination strategies has been applied worldwide. The massive vaccinations could decrease the number of infections and hospitalizations if they generate sufficient memory responses. During trials with the BNT162b2 (Pfizer/BioNTech) vaccination, 73% of the individuals developed a T memory response, 12 weeks after their second vaccination (Naaber et al. 2021). Sahin et al. (2021) showed that both a de novo CD4 and CD8 T cell response against at least one of the studied S peptide pools was present in ~ 97% of the participants. This shows that this vaccination indeed causes a memory T cell response.

Dennehy et al. (2021) studied vaccinated hospital staff to observe differences between vaccinated (with Pfizer/BioNTech) and infected individuals. The CD4 T cell responses were studied up to 3 months post-vaccination. The percentage of individuals with a T cell response was comparable, as was the frequency of CD4 ${T}_{CM}$ cells in both groups. However, some differences were observed: The vaccinated group had a higher frequency of specific CD4 ${T}_{EM}$ cells compared to recovered COVID-19 patients (Dennehy et al. 2021). The CD8 T cell response was studied by Oberhardt et al. (2021) 80–120 days post-vaccination. The frequency of S-specific CD8 T cells was higher post-vaccination compared to post-infection, as was the CD8 ${T}_{EM}$ subset. Reversed, the CD8 ${T}_{CM}$ subset was higher post-infection. While the distribution in subsets differed between the groups, the expansion capacity and cytokine production of the S-specific CD8 T cells were similar (Oberhardt et al. 2021). It is likely that S protein-specific T cell responses are somewhat lower after a natural infection than after vaccination, due to competition with the responses to the epitopes from other SARS-CoV-2 proteins.

### Impact of memory T cells on reinfection

How protective are memory T cells in preventing a reinfection with SARS-CoV-2 virus? Best models that address this question are Rhesus macaque. McMahan et al. (2021) found no virus in BAL and only 20% of animals had detectable virus in nasal swabs after reinfection, which took place 0–4 days after the first infection, suggesting that considerable natural immunity developed in macaques, at least for a short period of time. The role of CD8 T cells in the immunity was studied through the use of anti-CD8 antibodies, 7 weeks after initial infection and 3 days before reinfection, which causes almost complete CD8 depletion. All of the animals treated this way had detectable virus in their nasal swabs and their peak viral loads in nasal swabs were higher compared to controls with a fully functional CD8 memory T cell response. However, the peak viral load in anti-CD8 antibody-treated animals was lower compared to naive animals, which shows the impact of different memory responses other than CD8 T cells that contribute to natural immunity (McMahan et al. 2021).

The protective effect of vaccination has been studied as well. To determine the effectiveness of two doses of the mRNA-1273 (Moderna) and Pfizer/BioNTech vaccines in humans, placebo-controlled clinical trials were performed, where 30,000 subjects were assigned placebo or vaccination. During the first 7 days after receiving the second injection, 9 vaccinated participants and 169 placebo subjects developed symptomatic COVID-19 (Baden et al. 2021; Polack et al. 2020). In total, severe COVID-19 was observed in 9 placebo subjects and 1 vaccinated subject (Polack et al. 2020) in ~ 120 days after vaccination with Pfizer/BioNTech. During the study period, 19 vaccinated individuals and 269 placebo receivers developed symptomatic COVID-19 and all 30 subjects that developed severe COVID-19 were of the placebo group in the Moderna study (Baden et al. 2021). It has now also become clear that absence of vaccination is strongly associated with hospitalization and disease progression (Tenforde et al. 2021). All in all, these results show that vaccinations, similar to the infection as studied animal models (DiPiazza et al. 2021; Corbett et al. 2020), protect humans against development of symptomatic COVID-19. However, it does not indicate whether the vaccinations were also effective against the development of asymptomatic infections.

An important difference between natural infection and intramuscular vaccination is the generation of ${T}_{RM}$ cells. Lapuente et al. (2021) show that ${T}_{RM}$ cells are present after intranasal vaccination, but not after normal intramuscular vaccination for other viruses. In this study, mice were immunized with combinations of intranasal and intramuscular vaccinations. As expected, the group that got two systemic immunizations had almost exclusively circulating T memory cells. A Pfizer/BioNTech mRNA vaccination followed by intranasal Ad5 boost, however, induced both circulating and tissue resident T cell memory. Upon challenge, all of the vaccinated mice were protected against viral replication, clinical signs of the disease, and mortality, while 7 out of 8 of the unvaccinated mice died due to the disease. Surprisingly, there was no significant difference between vaccination routes (Lapuente et al. 2021), which might suggest that the presence of ${T}_{RM}$ cells did not significantly influence the outcome in this study. Given the known importance of ${T}_{RM}$ cells in other viral diseases, more in-depth studies should be performed in order to determine the role of ${T}_{RM}$ cells for SARS-CoV-2 infection.

Clearly, there are differences in the T cell memory compartment developed after infection with SARS-CoV-2 or vaccination as summarized above. Even though the memory T cell subsets expanded due to these two exposures are different, both give considerable, but not 100%, protection against the occurrence of reinfections. Besides this, both strategies significantly reduce the disease severity when reinfections do happen. So far, the difference in memory T cell subsets generated have not been shown to make any significant difference in this protective capability.

## Longevity of SARS-CoV-2-specific T cell responses

**Fig. 2:**
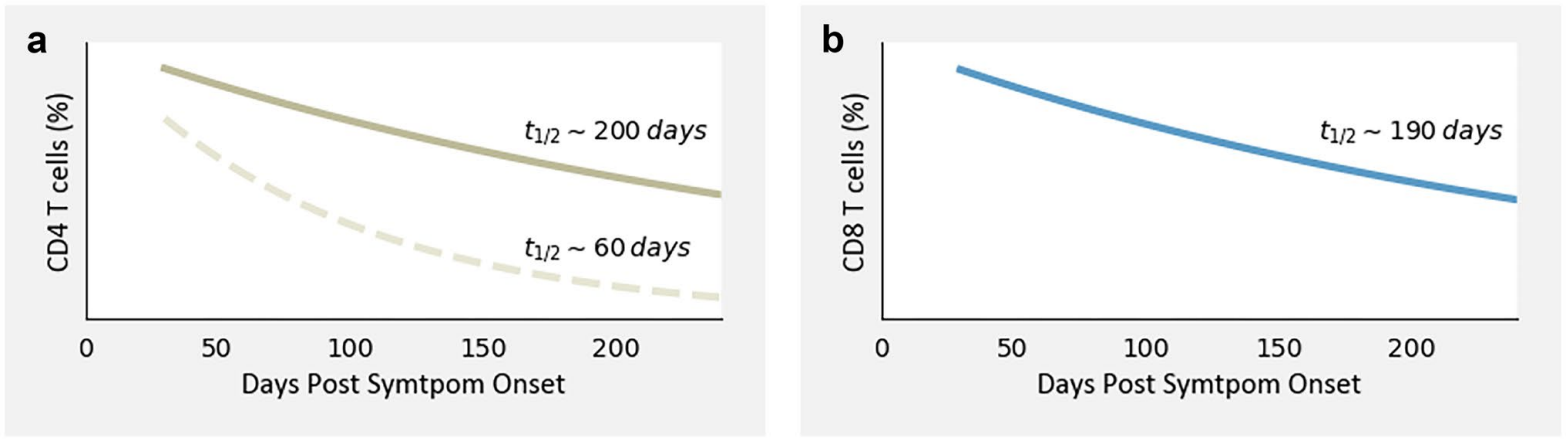
Visualization of decline of SARS-CoV-2-specific T cells. After initial infection with SARS-CoV-2 the number of T cells expand up to 1 month. Shortly after, the number of T cells starts to decline. The graph visualizes this decline and the dominant phenotype of T cells ~ 240 days post-symptom onset. To generate this graph we used a simple exponential function ($y\left(t\right)$= $\frac{1}{2}^{(t/T)}$, where $t$ stands for the half-life) with a half-life estimate of ~ 190 days for CD8 T cells, and two different estimates of ~ 60 days to ~ 200 days for CD4 T cells. These half-life estimates are taken from Cohen et al. (2021) and Dan et al. (2021)

Even 8 months post-symptom onset, memory T cells were still observed in 88% of recovered COVID-19 patients and 63% of healthcare staff (Havervall et al. 2022). However, the amount of the memory cells is not constant over this period of time (Cohen et al. 2021; Dan et al. 2021; Havervall et al. 2022). During the first month after symptom onset, the CD4 and CD8 T cells still expand, but afterwards, the number of cells declines with a half-life of approximately 200 and 190 days, respectively (Fig. 2, solid lines) (Cohen et al. 2021). Dan et al. (2021) estimated the half-life of CD4 memory T cells to be as low as 64 in paired samples (Fig. 2, dashed line for CD4 + cells). The difference between the two studies could partially be explained by the exclusion of non-structural proteins from the peptide pool within the study of Cohen et al. (2021), considering that Nsp3 was among the most recognised ORFs of the CD4 T cells (Dan et al. 2021). In line with these studies, Havervall et al. (2022) showed that the levels of memory T cells were significantly higher in the time period of 4 months post-infection compared to the time period of 5–8 months post-infection and beyond. Most importantly, they also observed that the memory T cell response at 5–8 months post-infection was not significantly different from the time period beyond 8 months (Havervall et al. 2022). Taken together, these studies suggest that the decline is mostly present during the first couple of months after infection while stabilization occurs later on.

Over time, most of the CD4 T cells are either of ${T}_{CM}$ or ${T}_{EM}$ phenotype, while the CD8 T phenotype shifts towards ${T}_{EMRA}$ cells (Cohen et al. 2021; Dan et al. 2021; Jung et al. 2021; Adamo et al. 2022). In light of longevity, the stem memory T cell (${T}_{SCM}$) subset is important to consider, as a small subpopulation of the ${T}_{SCM}$ cells is long-lived (half-life: ~ 9 years) and responsible for the maintenance of the T cell response over time (Costa Del Amo et al. 2018). Jung et al. (2021) studied the T cell subsets up to 10 months post-symptom onset. The frequency of ${T}_{SCM}$ cells of both CD4 and CD8 T cells increased until ~ 4 months post-symptom onset and then stabilized. The ${T}_{SCM}$ cells were stimulated and displayed robuste proliferation and differentiation towards a diverse set of T cell subsets (Jung et al. 2021). The ${T}_{SCM}$ cells were also retained over a period of at least 6 months after vaccination (Guerrera et al. 2021). The presence of ${T}_{SCM}$ cells, up to at least 10 months post-symptom onset, suggests that protective memory T cells have the capability to be retained long term. Moreover, Havervall et al. (2022) showed that the risk of reinfection remained reduced up to ~ 10 months post-symptom onset. This clearly suggests that memory T cells are not just present, but still functional in preventing disease, when potential stabilization of the number of memory T cells has occurred.

The protective capability of memory T cells developed upon vaccination is not necessarily the same as the protection acquired after natural infection. The effectiveness of Pfizer/BioNTech vaccination was determined over a period of 6 months to assess its long-term protective effect. Early on, the effectiveness of protection against symptomatic COVID-19 was 88% while this declined to about 47% at the 5-month mark. However, the effectiveness against hospitalization, and therefore disease severity, was preserved over the entire study period (Tartof et al. 2021). During another study, the vaccine efficacy against symptomatic COVID-19 was still at 83.7% around the 4-month mark (Thomas et al. 2021). This shows that vaccination is especially effective against severe disease in the first 6 months.

All in all, it can be concluded that protective memory does develop after SARS-CoV-2 infection and vaccination and it lasts up to at least ~ 1 year. Predictions for immunity on longer terms could be obtained through information on the related virus SARS-CoV-1 which caused a pandemic during 2003 (LeDuc and Barry 2004). In at least two studies, memory T cells were observed a long time after SARS-CoV-1 infection. Ng et al. (2016) stimulated PBMCs from three subjects (9–11 years after infection) and observed a small number of SARS-CoV-1-specific memory T cell responses against S, N, and M proteins. The number of responses in the studied subjects was significantly higher than in healthy individuals. All subjects had at least one N-specific CD4 and M-specific CD8 memory T cell response. Le Bert et al. (2020) observed memory T cells specific for the N protein of SARS-CoV-1, 17 years after SARS-CoV-1 infection, and the response pattern was significantly different in infected individuals compared to unexposed individuals. These results suggest that the memory T cells are indeed long lived after SARS-CoV-1 infection and that they do not represent cross-reactive T cells that have developed more recently due to infection with HCoVs. Thus, the number of SARS-CoV-2-specific memory T cells, which also respond strongly to S, N, and M proteins (Cohen et al. 2021; Sekine et al. 2020), might indeed be retained for a long time after stabilization around 8 months post-symptom onset and could provide a viable protection in the long term.

### Cross-reactivity against SARS-CoV-2 variants

So far, a couple of variants of SARS-CoV-2, that caused a new wave of infections, have been observed (Mistry et al. 2022). The new SARS-CoV-2 variants mainly, but not solely, contain mutations in the S protein (Mistry et al. 2022). To determine the effect of these mutations, Mazzoni et al. (2022) studied the wild-type-specific CD4 T cell response against different pools of peptides from the S protein: entire wild-type S and smaller regions of the S protein where mutations have occurred within variants. As expected, CD4 T cell responses for the entire wild-type S protein are significantly higher compared to the smaller wild-type regions where mutations are observed within variants. The majority of CD4 T cells respond to regions of the protein that have not been mutated. This suggests that only a minor fraction of S-specific CD4 T (less than 20% in general) cells will be influenced by the mutations (Mazzoni et al. 2022). These results are confirmed by Riou et al. (2022) who found that most of the CD4 T cell responses were specific against regions that had not been mutated in the Beta variant. They showed that none of the mutated epitopes was recognized by wild-type-specific CD4 T cells. However, they also observed that the mutated epitopes were poorly recognized by CD4 T cells developed after infection with the Beta variant. This could indicate that this region is not often targeted by CD4 T cells (Riou et al. 2022). Mazzoni et al. (2022) also found that mutations in the Beta and Delta variant did not have a significant impact on the CD4 T cell response. However, significantly lower responses were observed for the mutated Alpha and Omicron variants (Mazzoni et al. 2022). Geers et al. (2021) and Gao et al. (2022) obtained similar results for PBMCs that originated from individuals vaccinated with Pfizer/BioNTech. Geers et al. (2021) studied the effect of mutations in the Alpha and Beta spike variants on the CD4 T cell response from vaccinated individuals without prior infection. Again, there was no difference in CD4 T cell response between the wild-type and variant S peptides for either infection naive individuals nor those that had been through an infection before vaccination (Geers et al. 2021). Gao et al. (2022) reported that 91% of SARS-CoV-2-specific CD4 T cells from vaccinated individuals recognize Omicron, while only 84% of the specific CD4 T cells were responding in infected individuals. These results are in line with the significant differences observed by Mazzoni et al. (2022) for Omicron and the wild-type variant. However, the percentage of responding specific CD4 T cells for infected and vaccinated individuals was reversed in this study compared to the observations of Gao et al. (2022). Both studies do show that vaccinations, just like natural infections, will induce the development of T cells effective against the new variants.

The difference between T cell response against complete S protein and mutated regions was even more striking for CD8 T cell responses. Namely, the mutated regions were almost never targeted by the CD8 T cells (Riou et al. 2022). Redd et al. (2021a, b) studied the epitopes of SARS-CoV-2-specific CD8 T cells in more detail. They obtained 52 unique epitopes from five individuals with a median of ~ 42.5 days after diagnosis (Redd et al. 2021a). Only one of these 52 epitopes, restricted by HLA-A*24:02, had a mutation in the Beta variant (Redd et al. 2021b) and one, restricted by HLA-A*03:01 and HLA-A*11:01, was associated with mutations of the Omicron variant (Redd et al. 2021a). None of the mutations in the Alpha and Gamma variant overlapped with these CD8 T cell epitopes in these individuals (Redd et al. 2021b). The two affected epitopes were only recognized by a minority of CD8 T cells in the few individuals with a response against these epitopes (Redd et al. 2021a, b). Gao et al. (2022) studied the CD8 T cell response in a larger cohort for both infected and vaccinated individuals. For vaccinated individuals, similar results were obtained compared to those observed for CD4 T cells. However, the amount of CD8 T cells from infected individuals that could recognize Omicron (70%) was notably lower.

All in all, the studies of Mazzoni et al. (2022), Riou et al. (2022), Redd et al. (2021a, b) show that mutations only have a relatively small effect on the overall T cell response. Most of the specific T cells present in the population will still be effective against new variants. For example, ~ 90% of CD4 and CD8 T cell responses, from vaccinated individuals, against the S protein will still recognize the Omicron variant, while the cross-reactivity of CD8 T cell responses is lower after natural infection (Gao et al. 2022). However, T cell responses, developed during natural infections, will also be mounted against the other SARS-CoV-2 proteins, which are less mutated. Therefore, a comparison that solely relies on the response against the S protein does not directly show the difference in functionality.

The observation that mutations in the viral variants have only sparingly developed within T cell epitopes (Redd et al. 2021a, b) indicates that the VOCs have not yet escaped from the T cell response through the development of mutations. According to Minervina et al. (2022), the diversity in TCRs is important for the presence of cross-reactivity against the new variants. Moreover, polymorphism of HLA molecules could protect against the development of escape mutations, because escape from one specific HLA allele does not provide escape from the next host’s immunity. This could explain the observation that SARS-CoV-2 variants with escape mutations have not become the dominant strain (Minervina et al. 2022), especially if these escape mutations come at a fitness cost for the virus. In short, even though T cell escape mutations do occur, it does not seem to be a feature that is strongly selected for during SARS-CoV-2 evolution. Therefore, it seems that the obtained memory T cell response will be viable over a long period of time, even when the virus mutates and escapes from antibody responses.

## Discussion and conclusion

The literature reviewed above suggests that, overall, T cells are very important in the protection against severe COVID-19, at least when they respond relatively quickly to clear the virus. In doing so, they have the ability to prevent severe disease onset and potentially even prevent some reinfections. Due to intrinsic traits of cellular immunity, this protection is even viable in the long term. However, there are a number of remaining questions in the role of T cells during or protecting from a SARS-CoV-2 infection.

Having an early CD8 T cell response could help to predispose individuals to milder disease. However, in-depth studies into the role of bystander activation and more generally heterologous immunity before hospitalization are missing. Generally speaking, patients are submitted to the hospital days after onset of adaptive immune response (Huang et al. 2020) and it is difficult to trace back the exact time since infection or symptom onset. Upcoming studies could elaborate on the role of early T cell responses by studying subjects before and shortly after onset of symptoms. As it is very difficult to identify bystander cells via reliable markers, the dynamics of this heterologous immunity can best be studied by focusing on the specificity of TCRs.

. Results discussed in this review suggest that the presence of T cell memory, as a result of either vaccination or natural infection, reduces the risk of reinfection, or at least reduces the developing symptoms after rechallenge with the virus. However, the combined results from McMahan et al. (2021) and Soresina et al. (2020) suggest that while (CD8) T cell responses, even in absence of antibodies, are effective to promote recovery, they might not be able to completely prevent severe disease on their own. This is worrisome considering the observations that developed humoral responses do not give viable protection long term (Geers et al. 2021; Tang et al. 2011; Zhan et al. 2021). Therefore, the ability of memory T cells to prevent hospitalization, on their own, should be analyzed further, by comparing viral titers and severity of symptoms between subjects with and without detectable antibodies.

T cell responses do not only provide a protective effect, but could also be involved in tissue damage. In a subset of critically ill patients, overactivation and infiltration of (cytotoxic) T cells are associated with tissue damage (Gauchotte et al. 2021; Szabo et al. 2021; Xu et al. 2020). Consistent with the proposed timeline by Nienhold et al. (2020) and the role of cytokines in severe disease, increase in cytokine levels could promote overactivation after disease onset has already occurred. However, the exact cause of T cell overactivation has not been discovered yet. During future studies, the interplay between innate and adaptive immunity, such as the role of cytokines and dendritic cells in T cell activation, should be taken into account. By finding the cause of overactivation better treatment options can be devised to prevent tissue damage and fatal outcome. However, Nienhold et al. (2020) only studied a small number of deceased subjects and many other studies show that tissue damage could also have been caused independent of T cells (Reindeiro et al. 2021). In conclusion, especially considering the larger role of the innate immune system in tissue damage, the potentially harmful effects of activated T cells later on in disease should not overshadow their early protective effect in preventing the onset of severe disease.

Even though shorter lived memory T cells decrease in number following the infection, some stable amount of (stem cell memory) T cells can be retained years after initial development (Cohen et al. 2021; Dan et al. 2021; Guerrera et al. 2021; Havervall et al. 2022; Jung et al. 2021). For this novel virus, we do not yet have any data on the long-term stabilization in the number of memory T cells. Even the long-term retainment of SARS-CoV-1-specific memory T cells (Le Bert et al. 2020) does not give conclusive evidence for longevity of SARS-CoV-2 specific T memory cells. The diversity of T cell responses could theoretically maintain long-term immunity despite the SARS-CoV-2 variants, because complete escape from a diverse pool of T cells is not likely. Presently, mutations have only occurred in few T cell epitopes, but escape mutations that have a greater effect could arise in the future. Furthermore, if only a small proportion of T cells will be retained, as was seen in SARS-CoV-1 (Le Bert et al. 2020; Ng et al. 2016), the longevity might be limited due to novel mutations (Mazzoni et al. 2022). One strategy to increase the longevity of T cell responses is inducing a broader response against proteins that do not acquire new mutations as quickly as spike protein. It could, thus, be beneficial to expand the current vaccination strategy to include more proteins, such as the N and M proteins. T cell responses for these non-spike proteins were still observed a decade after SARS-CoV-1 infection (Le Bert et al. 2020; Ng et al. 2016).

Just like memory B cells, the development of memory T cells should be a cornerstone of studying the developed immunity. Although the exact protective effect of T cells in the absence of antibodies has not been thoroughly studied yet, results suggest that T cells alone could offer considerable protection. Combined with the longevity and the fact that no variants have accomplished complete escape from memory T cell responses (Mazzoni et al. 2022), T cell responses, in our opinion, give some hopeful perspective for a life after the pandemic. While we should remain vigilant of the virus, the amount of developed T cell responses, as a result of prior infection and (new) vaccinations, should be able to contribute to lower levels of hospitalization due to severe COVID-19 moving forward.
